# Childhood Obesity and Its Comorbidities in High-Risk Minority Populations: Prevalence, Prevention and Lifestyle Intervention Guidelines

**DOI:** 10.3390/nu16111730

**Published:** 2024-05-31

**Authors:** Ahmad Alkhatib, George Obita

**Affiliations:** 1 College of Life Sciences, Birmingham City University, City South Campus, Edgbaston, Birmingham B15 3TN, UK; 2 School of Health and Life Sciences, Teesside University, Tees Valley, Middlesbrough TS1 3BX, UK; v8050505@tees.ac.uk

**Keywords:** obesity guidelines, children and adolescents, non-communicable disease, lifestyle intervention, minority populations

## Abstract

The prevalence of childhood obesity and its associated comorbidities is a growing global health problem that disproportionately affects populations in low- and middle-income countries (LMICs) and minority ethnicities in high-income countries (HICs). The increased childhood obesity disparities among populations reflect two concerns: one is HICs’ ineffective intervention approaches in terms of lifestyle, nutrition and physical activity in minority populations, and the second is the virtually non-existent lifestyle obesity interventions in LMICs. This article provides guidelines on childhood obesity and its comorbidities in high-risk minority populations based on understanding the prevalence and effectiveness of preventative lifestyle interventions. First, we highlight how inadequate obesity screening by body mass index (BMI) can be resolved by using objective adiposity fat percentage measurements alongside anthropometric and physiological components, including lean tissue and bone density. National healthcare childhood obesity prevention initiatives should embed obesity cut-off points for minority ethnicities, especially Asian and South Asian ethnicities within UK and USA populations, whose obesity-related metabolic risks are often underestimated. Secondly, lifestyle interventions are underutilised in children and adolescents with obesity and its comorbidities, especially in minority ethnicity population groups. The overwhelming evidence on lifestyle interventions involving children with obesity comorbidities from ethnic minority populations shows that personalised physical activity and nutrition interventions are successful in reversing obesity and its secondary cardiometabolic disease risks, including those related to cardiorespiratory capacity, blood pressure and glucose/insulin levels. Interventions combining cultural contextualisation and better engagement with families are the most effective in high-risk paediatric minority populations but are non-uniform amongst different minority communities. A sustained preventative health impact can be achieved through the involvement of the community, with stakeholders comprising healthcare professionals, nutritionists, exercise science specialists and policy makers. Our guidelines for obesity assessment and primary and secondary prevention of childhood obesity and associated comorbidities in minority populations are fundamental to reducing global and local health disparities and improving quality of life.

## 1. Introduction

Childhood obesity is a major public health problem worldwide due to its rapidly increasing prevalence, the increased risk of early-onset non-communicable diseases (NCDs), the reduced quality of life of the affected children and its implications for adulthood morbidity [1]. The global prevalence of obesity among children and adolescents aged 5 to 19 years has quadrupled in the last 41 years, with an estimated rate of increase in the age-standardised body mass index (BMI) of 0.32 kg/m^2^ per decade (95% confidence interval, CI 0.23–0.41) for boys and 0.40 kg/m^2^ per decade (95% CI 0.30–0.50) for girls [2]. Up to 124 million school age children were estimated to have obesity in 2016, and up to 250 million are projected to have obesity by 2030 worldwide [2]. However, this projection does not show whether there is a disparity amongst population groups and whether some paediatric groups might be at a higher risk of obesity comorbidities than others.

At the global level, low- and middle-income countries (LMICs) experience a faster rate of rise in obesity prevalence than high-income countries (HICs). For example, the number of overweight children aged under five years increased by about 23% between 2000 and 2022 on the African continent, while the overall population obesity rate has increased 32-fold in Sub-Saharan African compared with 3.4-fold in western HICs [3]. Some even believe that childhood obesity prevalence is stabilising in some HICs [2,4,5], which makes LMICs’ prevalence even more alarming. Within the same western HICs, evidence shows that children from minority ethnicity communities are at a higher risk of childhood obesity than their white counterparts [6]. For example, the 2011–2012 National Survey of Children’s Health and the 1999–2014 National Health and Nutrition Examination Survey of 6–17-year-olds in the US showed that about 25% of black, Hispanic and American Indian/Alaska Native children were obese, compared to 12% among non-Hispanic white American children [7]. In England, the difference in obesity prevalence between Year Six children from the most deprived and the least deprived areas was 5% in 2006 compared to 13% in 2019, indicating a widening of disparities in childhood obesity prevalence [8]. Population disparities in childhood obesity prevalence, at both the global and local levels, necessitate characterisation of the prevalence amongst populations and identification of effective interventions within populations at a higher risk of obesity and its comorbidities.

Obesity is defined as a chronic disease in itself, and its comorbidities are defined as any NCDs concurrent with obesity, such as cardiovascular disease (CVD), type 2 diabetes (T2D), hypertension and non-alcoholic fatty liver disease. The association between obesity and NCDs has been repeatedly demonstrated in older adult groups, but recent reports have shown a continuous increase among children and adolescents [9,10,11,12,13]. For example, 79.4% of USA youth (young adults and children) with T2D were reported to have obesity, compared with 16.9% among those without diabetes [14]. Another analysis of electronic health records from the USA integrated health system showed that T2D prevalence varied from 4.5% in those with a BMI < 25 to 30.9% in those with a BMI > 40 [15]. Ethnic disparities in obesity comorbidities have also been reported, where African Americans had the highest rate of obesity and T2D (91.1% of their specific total), followed by Hispanics (75%), whereas the Non-Hispanic white ethnic group had the lowest prevalence, at 68.8% [14]. Despite this evidence of an increasing burden of NCDs among children, there is still inadequate reporting of obesity-associated comorbidities due to a lack of appropriate global surveillance systems [16]. Therefore, detailed characterisation of the disparities in childhood obesity NCD comorbidities could help in appropriating relevant preventative interventions at the global or local level.

Lifestyle prevention of obesity is well established among adults but less so amongst children. A recent secondary analysis of a Cochrane review of 153 studies for childhood obesity interventions found that most lifestyle interventions were implemented through education in schools, despite its limited effectiveness in preventing childhood obesity [17]. Little is known about how population disparities could explain the lack of obesity interventions’ effectiveness among such high-risk groups.

This narrative review aims to (i) characterise population disparities in the prevalence of childhood obesity and its comorbidities, (ii) identify effective intervention strategies amongst minority ethnicities, (iii) provide targeted guidelines to inform policy and practice on childhood obesity and associated comorbidity prevention.

## 2. Reducing the Disparity in Obesity Measurements

(a) BMI cut-off points globally in LMICs vs. HICs

Preventing childhood obesity and its comorbidities requires appropriate obesity assessment and the identification of high-risk individuals using practical and accurate measurement methods. The World Health Organisation (WHO) defines overweight and obesity based on BMI cut-off points (≥25 kg/m^2^ for overweight and ≥30 kg/m^2^ for obesity) to indicate the excessive accumulation of adipose (fat) tissue in the body, which impairs health [18]. In children, overweight and obesity are typically assessed using the International Obesity Task Force (IOTF)’s recommendation to use BMI percentiles for age and sex and BMI standard deviations (SDs), which depend on standard growth data for children [18,19,20].

However, neither the BMI nor its percentiles or SDs take into consideration ethnic differences in body fat accumulation and distribution [21]. For example, Asian populations have a 2–3 kg/m^2^ lower BMI than Caucasians for the same body fat percentage (%BF) [22], which puts them at an increased cardiometabolic risk if the same BMI is used. A multiethnicity large cohort study in children aged 7 to 13 years in Mauritius recommended lowering the WHO-defined BMI obesity and overweight cut-off points by 4·6–5·9 units in South Asians compared with Caucasian children [23]. Hence, lowering the BMI cut-off points has been recommended in LMICs, especially for Asian and South Asian populations, to a BMI of ≥23 for overweight and ≥25 kg/m^2^ for obesity [24,25]. Several Asian health authorities, mostly from LMICs, now adopt a more appropriate obesity BMI cut-off point of 25 kg/m^2^ [26]. For example, the 5.5% obesity prevalence in India estimated using the standard 30 kg/m^2^ BMI cut-off point, was increased to 40.3% when the lower 25 kg/m^2^ ethnicity-specific cut-off was used [27].

(b) BMI cut-off points within HIC minority populations

HIC cohorts such as those of multi-ethnic children from the UK showed that BMI underestimated fat mass (FM), estimated by the deuterium dilution method, in South Asians, suggesting positive BMI adjustments of +1.12 kg/m^2^ (95% CI: 0.83, 1.41 kg/m^2^; *p* < 0.0001) for boys and +1.07 kg/m^2^ (95% CI: 0.74, 1.39 kg/m^2^; *p* < 0.0001) for girls [28]. The same study found that BMI overestimated FM in black African children, suggesting negative adjustments, but this was complex, as it varied by age group and range. The required adjustment varied between −0.13 kg/m^2^ (boys) and −0.12 kg/m^2^ (girls) in 10–12-year-olds and −5.52 kg/m^2^ (boys) and −5.06 kg/m^2^ (girls) in 4–6-year-olds [28]. Another large cross-section study of an English cohort of children recommended ethnicity-specific BMI cut-off points for obesity based on type 2 diabetes risks of 30, 28.1, 26.9, 26.6 and 23.9 kg/m^2^ for Caucasian, black, South Asian, Chinese and Arab populations, respectively [29]. Among a New Zealand cohort of 2-year-old children, Buksh et al. (2017) reported that for a BMI of the 5th centile in boys, the FM/FFM ratio for Pacific children was 0.22, whereas for Indian children, it was 0.27, translating to a 23% lower FM/FFM ratio in Pacific children compared with Indian children (*p* = 0.03). They concluded that the standard BMI cut-off points are not suitable for assessing childhood obesity or its cardiometabolic comorbidities in multiethnic New Zealand, and that there is a need for ethnic specific cut-off points [30].

Nonetheless, the ethnic minorities within HICs are very diverse, which makes it challenging to adjust an ethnicity-specific BMI cut-off point for each group. For example, minority populations in the in USA include Asian Americans, Hispanic Americans, Mexican Americans and Indian Americans, with specific childhood obesity prevalence challenges. African American girls are reported to have doubled obesity rates compared to Non-Hispanic white groups, whereas Asian American girls have a prevalence three times lower than their white counterparts [31]. In New Zealand, childhood obesity rates are 35% and 17.8% for Pacific and Māori communities compared with 6.6% in Asian communities, and 10.3% in Europeans/Others [32]. Given the diversity within the minority ethnicity communities in western HICs, it may not be inappropriate to recommend single cut-off points for entire ethnicity groups, and it is also very complex to develop and apply cut-off points for each ethnic group.

Regardless of ethnicity, the BMI’s validity had been questioned in both children and adults due to its poor predictability of obesity and associated cardiometabolic risk among USA children [32] and its invalid anthropometric limitation in assessing an excessive %BF or FM [31]. Despite those known limitations, several HICs continue to use standard BMI cut-off points, resulting in minority ethnicities in those countries being missed from obesity classifications, with a consequent increase in disparities in obesity and associated cardiometabolic disease prevalence [33].

We recommend ethnicity-specific BMI cut-offs (Table 1) as useful for indirect estimation of excessive adiposity in children, adding at least one direct assessment method as listed below (Table 1).

(c) Direct obesity assessments to mitigate minority ethnicities’ disparities

Ethnic differences in the estimation of excessive adiposity may be less prominent when more accurate, direct methods are used to assess adiposity. The most common methods include DEXA, BIA, magnetic resonance imaging (MRI), computed tomography (CT), computed tomography body composition (CTBC), air displacement plethysmography (ADP), whole-body potassium counters (WBKCs), the isotope dilution method (hydrometry) and underwater weighing. There are also obesity/overweight assessment methods validated for infants’ %BF and %FFM, such as Pea Pod and ADP, but reference values for different ethnic groups are scarce [34].

DEXA is considered a gold standard for body composition assessment. It uses a three-component model to estimate fat, FFM and bone mineral density [35]. DEXA’s use has been shown to be feasible in assessing the body composition of large cross-sectional cohorts in LMICs (e.g., 1640 Indian school children aged 7–17 years) and HICs (217 Dutch children aged 10–11 years) [36,37]. Within 663 USA overweight and obese children, DEXA provided a significantly better prediction of %BF in both boys and girls than BMIs, especially in children aged 9 years or less [38]. Reference DEXA curve data for children showed a range of %BF values of 12.5% for the 3rd percentile and 51% for the 97th percentile. However, a cut-off value of 21%BF has been associated with obesity-related comorbidities of hypertension and T2D regardless of ethnicity [39,40,41,42].

Similarly, the less expensive BIA method, which uses two components (%BF and FFM%), has now become widely available in the form of standing scales and hand grip sensors and could be easily used in childhood obesity surveillance [43,44]. Examples of large-scale obesity screening in children populations using BIA include 12,426 Chinese school children aged 7–17 years measured using BIA bipedal devices within community healthcare centres [45], 2093 Czech children aged 6–11 years measured via BIA for %BF and FFM at their schools [46] and 272 Italian children in two primary schools [47]. In an English cohort, BIA (using a standing BIA scale) was a better predictor of the %BF among 194 UK South Asian children aged 8.47 ± 0.50 than BMI [48]. These studies demonstrated that BIA is a cost-effective measure of body composition feasible for large cohorts of children from multiple ethnicities within school or community settings.

DEXA and BIA have both been shown to be feasible in large population surveys, mostly in HICs [49,50,51,52], and thus we recommend their use in obesity surveillance among high-risk children, especially minority groups. However, in the less developed health systems of LMICs, these can be expensive and less practical when used at the population level. Instead, indirect measures of adiposity such as skinfold thickness, waist circumference (WC), the waist-to-hip ratio (WTR) and the waist-to-height ratio (WtHR) are good predictors of obesity-related metabolic risks. For example, compared with a child at the 10th percentile in terms of WC, a child at the 90th percentile was estimated to have higher LDL cholesterol (0.17 mmol/L), triacylglycerol (0.11 mmol/L) and insulin (6 mmol/L) and lower HDL cholesterol (−0.07 mmol/L), independently of BMI, race or sex [53]. Novel indirect anthropometric obesity indices such as wrist, neck, hand, calf, ankle, chest, lower arm, upper arm and hip circumferences, and finger ratios, have also been used to predict subcutaneous and abdominal fat. Those indices were also associated with cardiometabolic and obesity comorbidity risks [54]. However, their use is limited by skilled technician’s requirements, extensive training needs, and invasiveness [55].

We recommend using indirect techniques when resources are limited, as in many underdeveloped health systems in LMICs. Technological advances have made it feasible to access complex obesity assessment methods such as BIA and DEXA using simplified mobile, near-infrared, laser and motion sensor-based devices, which can still provide direct measures of adiposity detection for childhood obesity and its comorbidities in LMICs and therefore should be explored further [56,57]. Health infrastructure support to improve the research on surveillance and obesity interventions in LMICs is required. The available evidence suggests that surveillance of overweight and obesity, alongside early detection of obesity comorbidities, especially cardiometabolic risks in minority ethnicity children, require a multiple assessment approach.

**Table 1:** Anthropometric cut-off points for assessing overweight, obesity and body composition in high-risk children, including ethnic minority groups.

Childhood Obesity Assessment Method	High-Risk Population Type	Cut-Off for Overweight	Cut-Off for Obesity	Feasibility
BMI	Ethnic Asian and South and Central American children aged 2–18 years	23 kg/m^2^ or ≥85th to <95th percentile for age and sex [25,58]	25–27 kg/m^2^ or ≥95th percentile for age and sex [25,58]	All settings, physical and remote
	Other high-risk population groups, 2–18 years	25 kg/m^2^ or ≥85th to <95th percentile for age and sex [19,59]	30 kg/m^2^ or ≥95th percentile for age and sex [19,59]	
%BF using BIA	All paediatric populations, including high-risk ethnic minority children, 5–18 years	Boys: 19–20% Girls: 22–35% Also ≥85th–<95th percentile for age and sex [60]	Boys: 21–24% Girls: 24–35% Also ≥95th percentile for age and sex [60]	Feasible in primary healthcare settings, schools, sports and community centres, especially low-cost standing or handgrip scales
%BF and bone mineral density using DEXA	All paediatric groups, including high-risk ethnic minority children, 3–18 years	Boys: 18–23% Girls: 20–34% [61]	Boys, 3–18 years: 24–36% Girls, 3–18 years: 26–46% [61]	Suitable in well-controlled clinical and lab settings when a highly skilled technician is on site. Less accessible and expensive.
WC	All paediatric populations, including high-risk minority population groups, 6–18 years	≥90th percentile for age and sex [62]		Feasible in clinical settings, schools and specific centres in the community, but requires good skills, is time-consuming and may be problematic in some cultural settings.
WtHR	Asian, African and South American, 6–18 years	≥0.46 [63,64]		Feasible in clinical settings, schools and specific centres in the community, but requires good skills, is time-consuming and may be problematic in some cultural settings.
	Caucasians, 6–18 years	≥0.5 [63,64]		
Other anthropometric assessments: NC MUAC WrC HC Planter foot arch height	All paediatric populations, including high-risk minority population groups, aged 5–17 years.	NC: A range of 27–38 cm for boys and 27–33 cm for girls 6–17 years in some population groups (no standard recommendation for children) [65,66,67] MUAC: 25 cm for children aged 9–11 years [68] WrC: 97th percentile for predicting metabolic syndrome in children [69] HC: Correlates with %BF, but no data on reference or cut-off [54] Lower planter foot arch height is associated with obesity [70]		There is limited information about these anthropometric measures; therefore, more reference data are needed to validate such anthropometric tools.

BMI, body mass index; %BF, percentage of body fat; BIA, bioelectrical impedance analysis; DEXA, dual-energy X-ray absorptiometry; HC, hand circumference; MUAC, mid-upper arm circumference; NC, neck circumference; USA, the United States of America; WC, waist circumference; WtHR, weight-to-height ratio; WrC, wrist circumference. Reported cut-offs are available for BMI for age groups of 2 to 18 years [19,25,58,59], whereas BIA cut-off is for 5 to 18 years [60], DEXA 3–18 years [61], WC 6–18 years [62] and WtHR 6–18 years [63].

## 3. Understanding the Disparity in the Prevalence of Childhood Obesity and Its Comorbidities

The disparity in the prevalence and prevention of childhood obesity presents a new challenge for public health due to a continuous widening gap among different population groups, ineffective prevention strategies and emerging obesity comorbidities among paediatric populations.

(a) Disparity between HICs and LMICs

While an obesity pandemic manifested in HICs gradually for over a century [71], the rate of change in LMICs has been much faster, where it took less than 20 years to reach HICs’ levels of childhood obesity prevalence [72]. For example, evidence from primary care electronic health records in England shows that the odds of overweight and obesity in children increased by 8.1% per year between 1995 and 2003 (95% CI: 7.2% to 8.9%) compared with 0.4% (−0.2% to 1.1%) between 2004 and 2013 [73]. Whereas, in Sub-Saharan Africa, the number of overweight or obese children nearly doubled from 5.4 million in 1990 to 10.3 million in 2014 [74]. A similarly high rate of rise was observed in India, where pooled data showed an estimated prevalence of childhood excessive weight of 19.3% in 2010 compared to the 16% reported in 2001 (two-sample z-test, *p* < 0.01) [75]. Furthermore, prevention programs for global obesity and related comorbidities, including hypertension and T2D, have been implemented in HICs for several decades longer than in LMICs [76].

Explaining the differences in the rate of increases in childhood obesity prevalence between HICs and LMICs is complex. Some attribute this disparity to a better awareness on lifestyle prevention of obesity risk within HICs, in contrast to a sudden increase in intense marketing of and access to energy-dense foods in LMICs [77]. The nutrition transition to energy-dense and western-type poor nutritional habits within LMICs has been explained by a global trading environment that is affecting lifestyle behaviours rapidly [78]. Examples include a shift away from traditional foods, which has led to the popularity of calorie-dense diets, with added sugar; declining PA levels due to a shift from manual labour to the use of machinery and decreased active transport, such as walking [79]. In a multi-site survey involving 68 LMICs and over 180,000 children aged 12 to 15 years, it was found that only 9.1% of the children met the WHO-recommended PA levels, and most categorised as sedentary [80]. Table 2 below further illustrates the low rates of PA, increased sedentary behaviour and diet in LMICs. Despite the increasing prevalence of obesity in LMICs and increased obesogenic behaviours, country-wide lifestyle prevention strategies could still be successful in preventing obesity and related comorbidities such as diabetes among high-risk populations [81] and may be implemented within LMICs and among children with obesity.

While research examining family diet and meal characteristics in minority ethnicity populations in HICs is scarce, such characterisations provide stakeholders with useful information for helping families adopt a healthy diet, including on the consumption of fruit and vegetables and reducing screen time at family meals. In a study of the association between the healthfulness of foods served at family dinners and child dietary intake among 120 primarily low-income minority families, Trofholz et al. (2017) found that nearly half of the meals had added sugar, few meals served fruit and most of those meals were high in sodium and dairy components [82]. This suggests that the foods served at family meals determine the dietary quality for children. Therefore, the engagement of families in childhood obesity prevention interventions among minority ethnicity pollutions is vital for their effectiveness.

**Table 2:** Illustrations of prevalence levels of PA, healthy diet and sedentary behaviours among children LMICs.

Study, Age Group and Setting	Physical Inactivity Prevalence	Healthy Diet Prevalence	Sedentary Behaviour Prevalence
Godakandaate et al., 14–15 years, Sri Lanka [83]	87.5% < 2 days/week participation in PA	36.4% adequate fruit consumption; 66.9% adequate vegetable consumption	96.6% had a total sedentary time of ≥4 h/day
Peltzer and Pengpid, 13–15 years, ASEAN countries [84]	80.4% were physically inactivity	-	33.0% were sedentary
Ma et al., 12–15 years, LMICs [80]	Only 15.3% achieve WHO-recommended PA levels	-	64.6% were sedentary
Xu et al. (2020), 12–15 years, LMICs [85]	Only 15.2% achieved PA recommended levels	-	34.6% were sedentary
Khan, Khan and Burton (2022), 12–17 years, Afghanistan [86]	86.0% were not sufficiently active (PA < 7 days/week of ≥60 min/day)	-	31.0% were sedentary (sitting times ≥ 3 h/day)

ASEAN, Association of Southeast Asian Nations; LMICs, Low-and Middle-Income Countries, PA, Physical activity; WHO, World Health Organization.

The reported childhood obesity prevalence rates among LMICs vary from 0.3% among boys in Uganda to a high of 15.7% among girls in Nauru [87]. Many LMICs such as Bangladesh, Nepal, India and many countries in Sub-Saharan Africa face a double burden of obesity alongside undernutrition among children [88]. Data from demographic and health surveys/national health surveys of 15–18-year-old girls conducted in over 53 LMICs showed that 38% of countries had both overweight and underweight prevalences of greater than or equal to 10%. South Asia had the highest rate of underweight prevalence, increasing annually by 0.66%, whereas Latin America had the highest rate of overweight, with an annual increase of 0.5% [89]. Such a high prevalence of early malnutrition and later exposure to obesogenic environments are likely to lead to obesity in later life. The rising trend of a double burden of undernutrition and childhood obesity in LMICs requires that strategies to reduce childhood obesity must be balanced with improving nutrition in children at risk of undernutrition. Thus, a multi-pronged, individualised approach that addresses both obesity and underweight is needed in LMICs.

Obesity comorbidities in children were not heard of in the last century, but now they have risen alarmingly globally given that 2.1 billion people are considered obese (WHO 2018). Our recent systematic review provided evidence of disparities in the prevalence of emerging childhood obesity-related NCD comorbidities both globally and within HICs [90]. Among 651,659 children in the studies we analysed, there was a significantly higher prevalence of childhood obesity comorbidities in LMICs than in HICs (hypertension: 36% vs. 13%, metabolic syndrome: 27% vs. 6%, non-alcoholic fatty liver disease: 48% vs. 23% in LMICs vs. HICs, respectively). Global disparities were identified between regions, where Asia had the highest prevalence of childhood obesity-related hypertension (38.6%), followed by South America (25.3%) and Europe (20.1%). Within HICs, disparities were found in that minority ethnicity communities had higher childhood obesity and related comorbidity (75% vs. 65%), dyslipidaemia (12% vs. 9%), abnormal liver function (52% vs. 44%) and acanthosis (58% vs. 20%) levels than their white counterparts [90]. Therefore, interventions to address children and their outcome measures should also address NCD comorbidities.

Additionally, most LMICs have a high burden of communicable diseases (CDs) compared to HICs, where LMICs contribute to 75% of the global mortality from NCDs, and 90% of deaths due to CDs occur in Sub-Saharan Africa [91,92]. This double burden poses a threat to an effective response to childhood obesity because limited resources are prioritised for addressing CDs, while often neglecting the rapidly rising problem of childhood obesity and related NCDs [93]. Therefore, the gap in the prevalence of childhood obesity between HICs and LMICs could continue to widen due to the lower focus on childhood obesity and related NCDs in LMICs. This should be addressed by a tailored comprehensive strategy for the prevention of multiple long-term conditions, which may include NCDs and CDs.

(b) Disparity within HICs

There are wide gaps in the prevalence of childhood obesity within HICs based on factors such as ethnicity and socioeconomic status [94]. Evidence shows that within most western HICs, minority ethnicity groups have a higher prevalence of childhood obesity than their white counterparts [6]. In the USA, for example, data from the 2011–2012 National Survey of Children’s Health and the 1999–2014 National Health and Nutrition Examination Survey of 6–17-year-olds showed that about 25% of black, Hispanic and American Indian/Alaska Native were obese, compared to 12% among non-Hispanic white American children [7]. Similarly, in England, children from black ethnic groups have greater odds of being obese or overweight compared to white children (Odds Ratio of 1.7 and 1.4 for Black Caribbeans and Black Africans respectively) [94]. In England, the prevalence of obesity among children living in the most deprived areas is more than double that of those living in the least deprived areas [95]. Similarly, children from households with a low income and low education in the USA were reported to have about four times higher odds of obesity than children from households with a high income [7]. Therefore, in HICs, intervention for the prevention of childhood obesity should be designed to address disparities in risks, as well as the socioeconomic drivers of childhood obesity.

Table 3 below illustrates the ethnic disparities in the prevalence of childhood obesity in different HICs. It demonstrates that most ethnic minority groups, except for Chinese people in the UK and Non-Hispanic Asians in the USA, have a higher prevalence than their white counterparts. This suggests that ethnic minorities in western HICs are very diverse, with peculiar childhood obesity challenges. In some cases, the burden of childhood obesity in a minority ethnicity group is less than that of the majority white populations, for example, the Chinese community vs. the white population in the UK. The population disparities in the prevalence of childhood obesity and its comorbidities in HICs are therefore complex, but the overall pattern is that minority ethnicity groups are disproportionately affected. Therefore, there is need for a contextualised approach to the prevention of and interventions for obesity and its comorbidities within populations at higher risk.

## 4. Contextualising Prevention and Lifestyle Interventions in High-Risk Population Groups

It is widely accepted that primary prevention of childhood obesity and related NCDs requires a broader life-course approach, which examines intergenerational risk factors and addresses: pre-conception health; maternal and infants’ health, preschool children’s health, school-age children and adolescents’ lifestyle practices [98]. However, current evidence shows that despite universal prevention efforts by health authorities, the prevalence of childhood obesity and related NCDs continues to exponentially rise among high-risk populations groups, such as ethnic minorities in HICs, children from households of a low socioeconomic status in HICs and those in LMICs [88]. For example, the difference in childhood obesity prevalence between Year Six children from the most deprived and the least deprived areas in England widened from 5% in 2006 to 13% in 2019 [8]. Such disparities suggest that the lifestyle approaches currently deployed to address childhood obesity may have differential effectiveness between high-risk populations and the general paediatric population.

Lifestyle prevention of disease is well established across adult groups for a range of long-term conditions, including obesity, diabetes and cardiovascular disease [81,99,100,101]. However, some health authorities and funders still resist implementing lifestyle interventions in their populations due to their perceived implementation barriers and cost-effectiveness, with major negative consequences for high-risk groups, including children and minority ethnicities. For example, an NICE statement in 2011 concluded that the implementation of obesity-related lifestyle, PA and nutrition interventions in high-risk ethnic minority groups in England was not effective due to perceived cultural, religious and familial engagement barriers in high-risk ethnic minority populations [102]. Lifestyle characteristics apply to all populations that present with obesity, not only those considered minorities. Therefore, understanding why some population groups, especially those from minority ethnicities, who have a higher prevalence of obesity and its comorbidities, are hard to reach is crucial to implementing effective lifestyle interventions.

Contrary to the perception that a lack of health awareness causes minority ethnicities’ lack of engagement in lifestyle interventions, the existing evidence suggests other factors, especially the methods of engagement and cultural context. For example, Rawlins et al. (2013), in a qualitive study of healthy eating and PA in children aged 8 to 13 in a multi-ethnic community, found that awareness of key dietary messages and an emphasis on dietary variety and balance were high among the entire population, but for ethnic minorities, places of worship were key focal points for social support through which they could be fully engaged. Furthermore, our preliminary study in the North East of England showed that parents were fully aware of their children’s obesity and its comorbidities’ status but were less aware of the available preventative support from the National Health Service, especially lifestyle and weight management services [103,104]. Therefore, a dichotomy exists between health provision and end users, such as between what is offered by the health authorities and what minority ethnicities perceive as being offered. A more direct and engaging “bottom-up” approach is likely to be most effective in minority ethnicities within HICs.

Our recent systematic and meta-analysis of effective lifestyle interventions analysed over 26,000 children of minority ethnicities who underwent 6-month to 5-year lifestyle, nutrition and physical activity interventions. The question was to test whether such lifestyle obesity interventions were effective in this high-risk group. The results showed that multicomponent (modifying PA and nutritional behaviours) interventions implemented under controlled settings, such as in schools and under parental supervision, were more effective in preventing childhood obesity than those that were conducted remotely among high-risk population groups in HICs [105]. We also showed that parental and community engagement in the co-design of contextualised preventative interventions for childhood obesity works better in such communities. However, the diverse high-risk minority ethnicity communities in HICs respond differently to various intervention approaches. For example, Hispanic children tend to respond to interventions that are predominantly educational [106,107,108], whereas Chinese American children respond well to interventions that are online and computer-based, but these are much less effective in African American children [109,110,111]. Effective approaches in high-risk populations involve direct engagement of children and parents in the implementation of appropriate healthy diet and PA interventions, rather than distribution of educational materials and classroom education [112]. As parents and the community play prominent roles in the daily life of children, including influencing their dietary habits and PA, interventions that include parents are more effective than those purely based in school settings [113]. Therefore, implementing effective lifestyle interventions in high-risk population groups in HICs should be contextualised according to their engagement.

On the other hand, the implementation of effective childhood obesity prevention interventions in LMICs may be hampered by weaknesses in health systems and inequitable access to health services due to the socioeconomic inequality within LMICs [88]. Therefore, locally driven prevention approaches should take into consideration these systemic factors and target childhood obesity prevention within the overall strategy of improving the health and wellbeing of children. For example, we have emphasised contextualised obesity assessment methods for BMI and the use of BIA and DEXA for %BF and bone density where possible (Table 1). We also suggest adding multiple secondary disease outcomes using cost-effective tools to measure hypertension, cardiorespiratory fitness and nutrition status among high-risk communities. Strategies for lifestyle prevention in LMICs are reviewed in detail in Alkhatib et al. (2021) [114].

High-risk ethnic minority populations in western HICs with a high prevalence of childhood obesity require lifestyle interventions that include culturally contextualised PA and practical healthy nutritional guidance based on available/affordable foodstuff. Supervised but entertaining play activities of moderate to vigorous intensity are more sustainably effective among high-risk ethnic minority children than gym instructions would be [115,116,117,118]. Depending on the setting, such interventions can be delivered by sports trainers, health professionals, trained Physical Education (PE) teachers or volunteers with the appropriate skills [119,120]. The implementation of combined lifestyle intervention components (PA and nutrition) in school and home settings is effective as long as teachers are adequately trained and parents are involved in culturally appropriate PA and healthy nutrition promotion activities [121,122]. As in every population group, longer durations of interventions of more than 6 months provide more sustained effectiveness in minority ethnicity populations [123].

**Figure 1:**
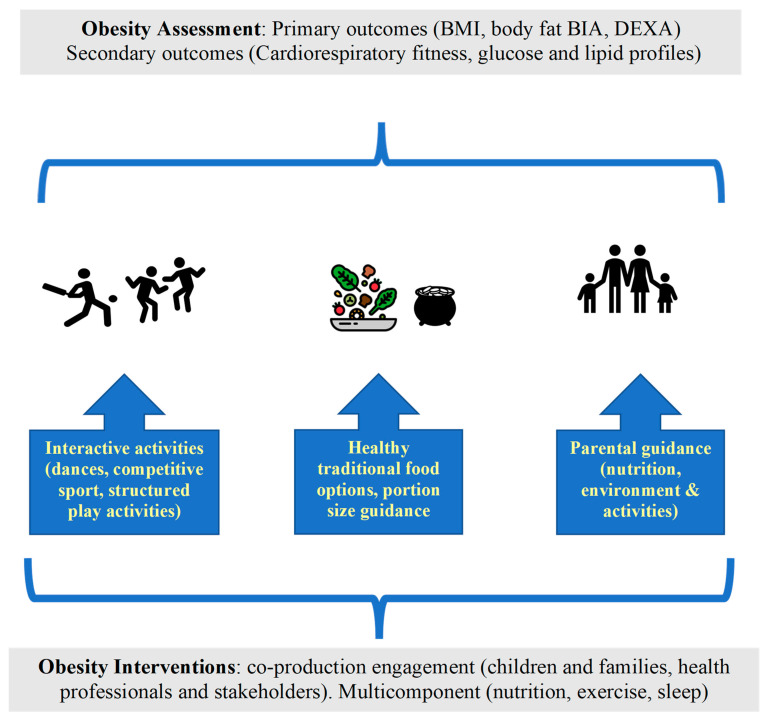
Guidelines for childhood obesity and comorbidity prevention in high-risk minority populations.

The components of lifestyle obesity intervention in minority ethnicity groups are similar to those applied to the general population, but in minority ethnicity populations, cultural contextualisation, co-design and co-production should be more emphasised (Figure 1). Recent models of co-design and co-production, especially those involving an inter-disciplinary team, are useful [124]. Our recommendation is that an inter-disciplinary team devising a lifestyle intervention related to childhood obesity in minority ethnicity groups should include members of these minority ethnicities, especially qualified specialists from such groups, including policy makers, community stakeholders, health professionals, exercise scientists, sport trainers, nutritionists and dietitians. Members of minority ethnicity groups should be involved in co-producing lifestyle interventions components, including: the baseline assessments, the relative focus on the nutrition versus exercise, the appropriate settings (school, community, places of worship, home-based), the appropriate intervention durations, and the evaluation and policy.

## 5. Conclusion and Recommendations

Childhood obesity disparities in the prevalence and interventions across different populations necessitate evidence-based guidelines. Public health policy and practice related to childhood obesity and associated comorbidity prevention in minority populations should consider the following recommendations (Table 4).

## Figures and Tables

**Table 3:** The prevalence of childhood obesity by ethnicity in selected HICs.

Country	Ethnic Group	Childhood Obesity Prevalence
**USA** (children 2–19 years) [31]	Non-Hispanic white	Boys 17.4% Girls 14.8%
	Hispanic	Boys: 28.1% Girls: 23%
	Mexican Americans	Boys: 29% Girls: 24.9%
	Non-Hispanic Asian	Boys: 12.4% Girls: 5.1%
	Non-Hispanic black	Boys: 19/4% Gils: 29.1%
**UK** [96]	Asian	Reception year (4–5 years): 10.1% Year 6 (10–11 years): 27.6%
	Black	Reception year (4–5 years): 16.2% Year 6 (10–11 years): 33.0%
	Chinese	Reception year (4–5 years): 4.5% Year 6 (10–11 years): 17.7%
	Mixed	Reception year (4–5 years): 10.7% Year 6 (10–11 years): 25.2%
	White	Reception year (4–5 years): 9.7% Year 6 (10–11 years): 21.8%
	Other	Reception year (4–5 years): 11.9% Year 6 (10–11 years): 28.5%
**New Zealand** [32]	European	10.3%
	Māori	17.8%
	Pacific	35.3%
	Asian	6.6%
**Australia** (children 2–14 years) [97]	Non-indigenous	25%
	Indigenous	30%

**Table 4:** Summary of recommendations for lifestyle interventions for childhood obesity and comorbidity prevention among high-risk populations.

Number	Recommendation
1	Reducing population disparities in childhood obesity and its comorbidities based on targeted prevention to reduce prevalence, accurate measurements and contextualised interventions
2	Basing childhood obesity interventions amongst minority ethnicities jointly on primary and secondary disease outcomes associated with obesity
3	Using “Personalised Obesity Prevention” so that effective interventions in high-risk population groups are contextualised, especially in terms of the combination of nutrition and physical activity, parental involvement and cultural context
4	Co-designing and co-producing effective community-based nutritional and physical activity strategies by involving healthcare professionals, nutritionists, exercise scientists and policy makers
5	Appropriately engaging with high-risk population groups to reduce the mismatch between what is being offered by healthcare authorities as “obesity prevention” and what is being perceived by minority populations as the end users.
